# Should Children Drink Water with Very Low Mineral Content? Implications of the Global Expansion of Water Filtration Systems and Relevance of Consumption of Water with Higher Mineralization Levels

**DOI:** 10.3390/nu18010103

**Published:** 2025-12-28

**Authors:** Cidália D. Pereira, Maria João Martins

**Affiliations:** 1 School of Health Sciences, Polytechnic of Leiria, 2411-901 Leiria, Portugal; 2 Centre for Innovative Care and Health Technology, Polytechnic of Leiria, 2411-901 Leiria, Portugal; 3 LSRE-LCM—Laboratory of Separation and Reaction Engineering-Laboratory of Catalysis and Materials, Polytechnic of Leiria, 2411-901 Leiria, Portugal; 4 ALiCE—Associate Laboratory in Chemical Engineering, Faculty of Engineering, University of Porto, 4200-465 Porto, Portugal; 5 Instituto de Investigação e Inovação em Saúde (i3S), Universidade do Porto, 4200-135 Porto, Portugal; mmartins@med.up.pt; 6 Unit of Biochemistry, Department of Biomedicine, Faculty of Medicine, University of Porto, 4200-319 Porto, Portugal

**Keywords:** water mineral content, natural mineral water, water filtration systems, children, bone, calcium

## Abstract

The consumption of water with very low mineral content (W-VLMC; water with total dissolved solids below 50 mg/L), despite limited and inconsistent evidence and the resulting knowledge gaps, has not been associated with health risks for the general population. However, certain population subgroups (those eating very unbalanced diets or avoiding certain foods, engaged in prolonged periods of fasting, and/or doing prolonged or strenuous exercise as well as pregnant or breastfeeding women) should be mindful of maintaining sufficient intake of all essential minerals through their food if regularly using this type of water as their main beverage. The rapid expansion of water filtration systems—often producing W-VLMC—creates a timely and valuable opportunity to advance research on the health implications of W-VLMC intake. As these systems become increasingly common in educational settings and homes, children represent a subgroup experiencing rising exposure to W-VLMC. Additional studies are needed to assess the health effects of such exposure from early childhood. A complementary yet contrasting perspective is that the use of water intended for human consumption—with stringent quality control standards—and natural mineral waters—inherently pure, thus eliminating the need for filtration—with higher mineralization in both types of water, may provide an additional dietary source of essential minerals, especially for all the population subgroups mentioned above.

## 1. Assessment of the Risk of Consuming Water with Very Low Mineral Content

In the context of European legislation, water intended for human consumption excludes natural mineral water and medicinal water, while spring water may fall under this definition depending on its mode of supply and regulatory classification, as these categories are governed by partially distinct legal frameworks [1,2]. Water with very low mineral content (W-VLMC), whether table, spring, or natural mineral waters, bottled or tap, has long been widely consumed, especially due to factors such as taste, perceptions of purity and safety, and, in some regions, water scarcity (the latter leading to the consumption of desalinated seawater, also bottled or tap). However, despite its wide use, robust data on its implications for human health remain limited [3,4,5,6,7,8]. Notably, the available evidence derives from heterogeneous study designs: epidemiological and public health studies have predominantly examined water intended for human consumption, whereas interventional studies have more often relied on natural mineral waters, owing to their stable and well-characterised mineral composition [9,10,11,12,13,14]. Here, W-VLMC refers to drinking water, either water intended for human consumption or natural mineral water, in which total dissolved solids (TDS) do not exceed 50 mg/L, a threshold commonly used to classify natural mineral waters [1,15].

In 2020, the German Federal Institute for Risk Assessment (BfR) assessed whether the consumption of W-VLMC (mineral, spring or table water) could present any risk to human health. According to BfR (in its opinion number 041/2020), despite the limited and inconsistent data, as well as the resulting knowledge gaps, it can be assumed that intake of this type of water does not cause long-term health risks for the general population when consumed in normal amounts alongside a balanced diet [6]. In fact, BfR considers drinking water as “one of the several day-to-day sources of mineral intake” [6] and therefore a balanced diet, in adequate amounts, would provide the needed mineral quantities.

These conclusions are consistent with some international guidelines, as neither the World Health Organization (WHO) recommendations nor the European Union Drinking Water Directive currently specify limits for calcium, magnesium, or hardness on health grounds [5,16,17]. Nevertheless, it should be mentioned that in older publications [3], the WHO issued recommendations for minimum and desirable or optimum contents of important minerals in drinking water (because of the consumption of demineralised water).

Although W-VLMC is not considered harmful to the general population, the BfR noted that certain consumer groups may require special attention if regularly using this type of water as their main beverage. Namely, people eating very unbalanced diets, those consciously avoiding certain foods (for example vegans or people with lactose intolerance), those engaged in prolonged periods of fasting, and pregnant or breastfeeding women should be mindful of maintaining sufficient intake of all essential minerals through their diet. Additionally, exclusive consumption of W-VLMC during prolonged or strenuous exercise (>1.5 h) was considered to be insufficient to maintain fluid and electrolyte balance and support optimal physical performance [6].

In order to determine whether new evidence published since the BfR’s risk assessment modifies or refines BfR’s conclusions, recent publications were reviewed through a PubMed search conducted on 10 October 2025, using the keywords “very low mineral water content consumption” and filtering only for publications dated after 1 January 2019 (a date chosen to ensure that no late 2019 and/or early 2020 publications were missing from the BfR’s risk assessment). Of the 41 articles retrieved, only four of them were found relevant: one review article, and three original research articles (with one already included in the BfR document) [18,19,20,21]. However, these three new publications do not in any way alter the recommendations established by the BfR in 2020 [6].

## 2. Simultaneity of the Increase in Water Filtration Systems Market and Consumption of Water with Very Low Mineral Content 

An excellent opportunity to deepen our understanding of the health impacts of consuming W-VLMC arises from the exponentially increasing use of water filtration systems worldwide (across North and South America, Europe, Asia-Pacific, Middle East, and Africa) [22].

This is because, depending on their characteristics, such systems can markedly (a) decrease the content of some specific ions, (b) decrease the content of some specific ions while increasing others, and/or (c) decrease the total mineral content of water. Water softeners replace the hard minerals in water [calcium and magnesium (and small amounts of iron)] with soft minerals (sodium or potassium). Ultrafiltration removes viruses, bacteria, and suspended solids, while nanofiltration and reverse osmosis also remove multi- and/or monovalent ions. Stricter water quality standards and growing awareness of contamination risks, such as heavy metals, pesticides, microplastics, and pathogenic microorganisms, as well as water scarcity, are driving this market’s expansion, with its global value projected to increase 66% from 2024 to 2032: from USD 15.88 billion in 2024, to USD 20.48 billion in 2028, and further up to USD 26.42 billion in 2032 [22,23,24,25].

The use of water softeners may mimic moving from a hard-water area to a soft-water area, resulting in a reduction in calcium intake equivalent to 17.5% of the recommended daily intake for adult males [26]. Depending on the water softener and on the water hardness, the use of these systems may dramatically increase the sodium concentration in the water being softened making it unsuitable for children under 8 years [27].

Given that many filtration systems produce W-VLMC, the global spread of their use amplifies exposure, underscoring the urgency of evaluating the long-term health implications of higher W-VLMC consumption.

This issue is particularly relevant in certain settings where these specific water filtration systems are increasingly used, and the resulting water is progressively more consumed by vulnerable population subgroups. One such example is schools and households [22]. In these environments, children—who spend much of their daily time there—constitute a particularly highly exposed, sensitive population. Although children require stricter water quality standards than adults, owing to not only physiological but also behavioural factors [28], the use of these specific systems to improve water quality leads to a reduction in the mineral content of the consumed water, which may be of concern during child growth and development.

## 3. The Use of Water Filtration Systems and Consumption of Water with Very Low Mineral Content by Children: (Main) Focus on the Impact upon Height Development and Bone Health

Data on the use of direct-drinking water systems targeting school-aged children have been published [18,28,29,30,31].

As abovementioned, the use of specific water filters decreases mineral intake by children [18,28,29,30,31], which will become particularly relevant when dietary mineral intake falls below the recommended levels. These studies present some experimental design limitations [6] and show inconsistent results [18,30,31]. In three of these four articles, the aim was to evaluate the impact of W-VLMC (available through direct-drinking water systems) on height development and/or bone health of those children [18,30,31]—a critical issue at this age. In all these studies, children were evaluated during 4 [18,29,30] or 5 years [31], and in two [18,30] a negative impact on height development/increase and bone health was observed, while no impact on height and height growth was found in one [31].

Huang et al. (2018) [30] conducted an eco-epidemiological study involving 29,884 schoolchildren (all living in an urban area) from 25 schools to evaluate the impact of the mineral content in the school filter-treated drinking water on their height development. Schools were organized into two groups according to the type of the direct-drinking water systems; therefore, in all schools, children did not drink the municipal tap water. The systems produced water with different conductivity values: in three schools, the water provided was similar to municipal tap water (one school used an ultrafiltration membrane combined with an activated carbon filter, while the other two used only an ultrafiltration membrane), whereas the remaining 22 schools provided water with much lower mineral levels (a reverse osmosis membrane combined with an activated carbon filter was used). Among other parameters, the statistically significant differences between the drinking water of the two groups were 14-, 12-, 5-, 6-, and 7-fold for calcium, magnesium, bicarbonate, hardness, and conductivity, respectively. Children exposed to the W-VLMC (second group) had reduced height and diminished height growth [30].

Huang et al. (2019) [18] conducted a retrospective cohort study involving 660 schoolchildren from 4 schools to evaluate the impact of the mineral content in the school filter-treated drinking water on their height development and biomarkers of bone remodelling. Similarly to what was described above, the children did not have access to municipal tap water, and the schools were organized into two groups according to the type of the direct-drinking water systems: in one group (one school) the filtration system used activated carbon combined with an ultrafiltration membrane, while in the other (three schools) reverse osmosis technologies were used [18]. As aforementioned, in the reverse osmosis vs. the ultrafiltration membrane group, the schoolchildren significantly ingested less calcium and magnesium from the water. Here, a significantly lower total calcium and magnesium intake was observed in the reverse osmosis vs. the ultrafiltration membrane group, despite similar dietary intake. It is of paramount importance to mention that no child (from either group) reached the recommended calcium and magnesium intake, highlighting the contribution of mineral water content to their total daily intake. W-VLMC was correlated with bone resorption activation (higher serum crosslinked C-telopeptide of type I collagen level), osteoblast inhibition (lower serum bone alkaline phosphatase activity), and reduction of bone mineral content as well as lower height increase [18].

In line, a statistically significant relationship between fractures in schoolchildren and naturally low calcium levels in the public water supply has been reported [32].

Just one article evaluated the effect upon children cardiovascular health, finding the consumption of W-VLMC a “new environmental concern risk” [29]. Evidence from studies including older individuals also supports the potential adverse cardiovascular effects of consuming water with reduced mineral content, underscoring the importance of further research focused on children [3,33,34]. Towns in England and Wales experienced higher levels of heart disease after their water supplies were softened [3]. The risk for ischemic heart disease increased with desalinated seawater (that is an example of W-VLMC) consumption among individuals aged 25 to 76 years [33]. Reduced magnesium intake resulting from the consumption of desalinated seawater may be associated with the increased all-cause mortality observed in acute myocardial infarction patients, approximately 63 years old, living in areas supplied with desalinated seawater, at both 30 days and one-year post-event [34].

Altogether, these findings raise the question of what the effects might be if exposure to W-VLMC (a) occurs right after birth (for example, when used to prepare infant powder formula), (b) continues for more than 4 or 5 years, or (c) happens throughout the entire life. As an example, for the former, metabolic acidosis, convulsions and brain oedema were observed in infants whose drinks had been prepared with bottled low-mineral water or distilled water [3,35].

## 4. Relevance of Water Mineral Content: (Main) Focus on the Impact of Higher Water Mineralization Levels upon Bone Health

Meeting the calcium daily needs is necessary for bone health through life: for bone growth and healthy development in infancy, childhood and adolescence, for maintenance in adult life, and for preventing and/or delaying bone mineral loss in the elderly [36].

Large population-based studies conducted in Europe and Asia have reported beneficial associations with bone mineral density and fracture risk, particularly in populations with low dietary mineral intake [37,38], as well as inverse associations between calcium and/or magnesium concentrations in public drinking water and cardiovascular mortality [39,40,41,42].

Although water intended for human consumption can contribute to cardiovascular and skeletal health, its mineral content is generally modest and highly variable, being influenced by local geology and treatment practices [43,44]. Natural mineral waters, by contrast, exhibit a much wider range of mineralization, from very low mineral content (TDS < 50 mg/L) to highly mineralized waters (TDS > 1500 mg/L), with calcium and magnesium concentrations ranging from a few mg/L up to several hundred mg/L. Importantly, as the mineral content of public drinking water fluctuates over time and across regions, the composition of natural mineral waters is remarkably constant due to their protected geological origin [1,15].

In addition, minerals from water (both from water intended for human consumption and natural mineral water) are highly bioavailable [3,4,9,36,45,46,47,48,49]. Depending on the calcium content and amount of water consumed, it can provide up to 100% of the daily calcium requirements [50,51,52,53]. Curiously, calcium-rich mineral waters (>150 mg Ca/L) have been considered as a good candidate source of this mineral [54]. In fact, consumption of natural mineral waters, rich in calcium and/or with high mineralization/bicarbonate levels, has been reported to have a strong beneficial impact on bone health [36,55,56,57,58,59,60,61,62,63]. We are unaware of studies in children. Although the regular consumption of calcium-rich natural mineral waters has been suggested as a non-pharmacological intervention for early menopausal bone mineral density preservation and reduction in long-term fracture risk [61], it should be noted that the intake of these waters is not recommended during bisphosphonate therapy, as they can interfere with the absorption of bisphosphonates [64].

A complementary, yet contrasting, perspective to Section 3 (The Use of Water Filtration Systems and Consumption of Water with Very Low Mineral Content by Children: (Main) Focus on the Impact upon Height Development and Bone Health) comes from Sangemini (Umbria Region, central Italy), where historical factors have led to two distinct sources of water being consumed by the local population. One sector of the population freely receives a natural mineral water, calcium-rich (Sangemini), whereas the remaining inhabitants have access to water with lower calcium content provided by public aqueducts. Despite sharing similar environmental conditions and lifestyles, women living in the first sector have significantly higher spinal bone mineral density than women living in the second, suggesting that long-term exposure to calcium-rich (natural mineral) water may contribute to improved bone health [56].

Cepollaro et al. (1996) [55] enrolled 45 early postmenopausal women (52.58 ± 1.99 years), in a prospective study, to randomly ingest, for 13 ± 1 months, 1 L/day of a high-calcium or low-calcium mineral water (providing 408 vs. 80 mg/L), being their total calcium intake 1510 ± 202 mg/d vs. 949 ± 181 mg/d, respectively. At the end of the dietary intervention with the two mineral waters, distal radius bone mineral density significantly decreased only in the women within the lower calcium intake group, with the two groups significantly differing in this parameter (although similar at baseline). The women with higher calcium intake had their osteocalcin serum levels decreased after 3 months [55].

Meunier et al. (2005) [57] observed, in a randomized double-blind placebo-controlled study including 152 postmenopausal women (around 70 years), that a daily supplement of 596 mg of Ca^2+^ through the (6-month) consumption of 1 L of calcium-rich mineral water (vs. 1 L of low calcium mineral water, providing 10 mg/L of calcium) was able to significantly lower serum parathyroid hormone as well as serum and urinary indices of bone turnover (osteocalcin and crosslinked C-telopeptide of type I collagen levels) in postmenopausal women with a low calcium intake (< 700 mg/d) [57].

Marino et al. (2023) [36] reported that a six-month intake of 2 L/day of a natural mineral water rich in calcium and bicarbonate by 120 perimenopausal women (mean age ≈ 47 years) resulted in: (a) improvements in calcium metabolism and bone health-related biochemical parameters; (b) restoration of mitochondrial energy-production pathways; (c) promotion of bone-mass accrual and collagen formation; and, (d) reductions in osteoporosis biomarkers. These effects were observed relative to an oligomineral, CO_2_-supplemented water containing 3.5-fold less calcium and 3-fold less magnesium than the mineral-rich water [36].

Among female dietitians (18–45 years), with adequate calcium intake, the consumption (1.5 L/day, for 28 days) of (a) an acidic, calcium-rich water had no effect on bone resorption, but (b) an alkaline, bicarbonate-rich, and similarly calcium-rich water led to a significant decrease in serum parathyroid hormone and C-telopeptides [63]. Interestingly, it has been discussed that high bicarbonate and/or magnesium content in calcium-rich waters may protect against calcium oxalate stone formation [53,65,66,67].

Furthermore, several review publications have highlighted the metabolic and physiological relevance [9,68,69,70] of consuming natural mineral waters with medium- or high-mineralization levels (waters with TDS > 500 mg/L) [1,15]. Beneficial effects have been disclosed upon lipid profile (total, LDL- and HDL-cholesterol as well as triglycerides), blood pressure and glucose homeostasis [9,69,70].

Additionally, it has been hypothesised that natural mineral waters could be chosen, according to their specific mineral compositions, to match hydro-saline requirements after different athletic performances [71].

## 5. Conclusions

Waters intended for human consumption are subject to strict regulatory standards and continuous monitoring to ensure the absence of harmful levels of pollutants and contaminants [2]. Under these conditions, domestic water filtration systems generally provide limited additional benefits in terms of safety, as the water is already compliant with established quality and health criteria. In addition, natural mineral waters are characterized, among other parameters, by their original purity due to their underground origin, which must be protected from all risks of pollution [1]. This eliminates the need for any type of filtration treatment before consumption and strengthens the message conveyed in this article. In addition, natural mineral waters have a specific chemical composition that remains stable over time (within the limits of natural fluctuation) [1]. Therefore, they can be selected according to individual mineral needs across the life cycle, as well as dietary and physical-activity contexts.

Given the increasing use of water filtration systems that produce W-VLMC, the lack of robust data on the long-term effects in children underscores the need for timely and comprehensive research. Moreover, cooking with W-VLMC may further increase mineral loss from foods, compounding the reduction in mineral intake that already results from W-VLMC consumption [72,73].

Finally, the consumption of sweetened beverages (soft drinks) should be considered in relation to the W-VLMC, as soft water is used in their production and has already been identified as a potential hidden variable contributing to the health risks associated with sweetened beverage consumption [74].

## Data Availability

Not applicable; No new data were created or analyzed in this study.
